# Optimization Milling Force and Surface Roughness of Ti-6Al-4V Based on Ultrasonic-Assisted Milling (UAM): An Experimental Study

**DOI:** 10.3390/mi14091699

**Published:** 2023-08-30

**Authors:** Qingqing Lü, Saiyu Yang, Liquan Yang, Erbo Liu, Guangxi Li, Daohui Xiang

**Affiliations:** 1 School of Mechanical and Power Engineering, Henan Polytechnic University, Jiaozuo 454003, China; 2696@pdsu.edu.cn (Q.L.); 2677@pdsu.edu.cn (G.L.); 2 Henan Province Engineering Research Center of Ultrasonic Technology Application, Pingdingshan University, Pingdingshan 467033, China; y494127523@163.com (S.Y.); 26101@pdsu.edu.cn (E.L.)

**Keywords:** micro-texture, milling force, roughness, surface morphology, ultrasonic-assisted milling

## Abstract

This study aimed to develop a longitudinal ultrasonic-assisted milling system to investigate the machinability of titanium (Ti) Alloy Ti-6Al-4V (TC4). Aiming at reduced milling force and enhanced surface quality, ultrasonic-assisted milling was investigated taking into account the following processing parameters: spindle speed (cutting rate) *n*, feed per tooth *f*_z_, milling depth *a*_p_, and ultrasonic amplitude *A*. A comparison was made with conventional milling. The results of univariate tests demonstrated that the ultrasonic amplitude had the most significant impact on the milling force along the *z*-axis, resulting in a reduction of 15.48% compared with conventional milling. The range analysis results of multivariate tests demonstrated that *a*_p_ and *f*_z_ were the dominant factors influencing the cutting force. The minimum reduction in the milling force in ultrasonic-assisted milling along the *x*-, *y*-, and *z*-axes was 11.77%, 15.52%, and 17.66%, respectively, compared with that in conventional milling. The ultrasonic-assisted milling led to reduced surface roughness and enhanced surface quality; the maximum surface roughness in ultrasonic-assisted milling was 25.93%, 36.36% and 26.32% in terms of *n*, *f*_z_, and *a*_p_, respectively. In longitudinal ultrasonic-assisted milling, the periodic “separation-contact” was accompanied by microimpacts, resulting in even smaller intermittent periodic cutting forces. Hence, regular fish scale machining mesh was observed on the processed surface, and the workpiece surface exhibited high cleanness and smoothness. The reasonable configuration of ultrasonic-assisted milling parameters can effectively improve the milling force and surface quality of Ti alloys and accumulate reference data for the subsequent machining process research.

## 1. Introduction

Titanium has been in use since the early 1950s. The first Ti alloy used was TC4, which is also the most widely used Ti alloy at present globally. Ti alloys are widely used in aerospace and other fields [1,2] because of their high specific strength and good heat and corrosion resistance [3,4,5], such as the disks and blades of aero-engine fans and pressurizers, which can resist high temperatures of up to 400 °C [6,7]. Ti alloys are ideal metal composites. However, Ti alloys have strong chemical activity and high thermal conductivity, which makes them prone to frictional heat during machining. They also have high hardness, strength, and corrosion resistance, which makes them very likely to result in cutting chip fracture and blade cracking during machining, thus significantly affecting the efficient and precise machining of Ti alloys [8,9]. Ultrasonic-assisted milling (UAM) is based on the conventional milling (CM) method with the integration of ultrasonic vibration, which can convert high-frequency oscillation electrical signals into mechanical vibration using an energy transducer, thereby achieving intermittent cutting effect [10,11]. This technique can reduce the cutting force and improve the machining quality [12,13,14,15]. Recently, many studies have been conducted on the mechanism of UAM for Ti alloys. Sun et al. [16] investigated the relationship between ultrasonic power and frictional coefficient during UAM through cutting simulation and experiments. The results showed that compared with CM, UAM with the assistance of rotational ultrasonic vibration, had very small high-frequency signals and a lower frictional coefficient, indicating that increasing ultrasonic power reduced the frictional coefficient. Pang et al. [17] proposed longitudinal–torsional ultrasonic vibration milling for the surface machining of Ti alloys, with the advantages of high efficiency and high quality; the machined surface showed good integrity and regularity. Zhao et al. [18] studied the influences of different directions of ultrasonic vibration on Ti alloys. The results showed that axial vibration was more conducive to the formation of micro-texture on surfaces and the reduction of the cutting force, wear, and running-in time under the control of cutting speed and ultrasonic amplitude. Wei et al. [19] compared the roughness *R*_a_ of the surfaces machined using UAM and CM and found that UAM could reduce the milling force and improve workpiece quality.

UAM has become a more accurate, efficient, and less damaging machining method than CM, as it can achieve a lower cutting force, lower frictional coefficient, and better surface quality, allowing its widespread application in many fields [20,21,22,23]. This study used UAM to investigate its machining performance for a Ti alloy by focusing on the triaxial milling force during machining and the Ti alloy surface integrity after machining. The trends of the milling force and surface integrity of the Ti alloy machined using UAM and CM were compared by changing machining parameters. The results showed the advantages and disadvantages associated with using UAM for the machining of Ti alloys and the machining performance pattern, providing references and technical support for Ti alloy machining.

## 2. Materials and Methods

### 2.1. Materials and Devices

The Ti alloy used in this study was TC4, which was a two-phase Ti alloy with 6% α-stabilized element Al and 4% β-stabilized element V. Al can improve the alloy strength at room temperature, whereas V can improve the alloy plasticity at room temperature and help the alloy maintain high comprehensive mechanical properties, thus avoiding the alloy brittleness at high temperatures [24,25]. The original material of titanium alloy in this test is ingot in an annealing state. The 15 mm × 6 mm × 8 mm sample is made by Wire cut Electrical Discharge Machining (WEDM) for the milling test. The sample processing method has the smallest impact on the surface mechanical properties of the material to ensure the accuracy of the milling test. The elemental composition and performance parameters of Ti alloys [26] are shown in Table 1 and Table 2, respectively.

**Figure 1:**
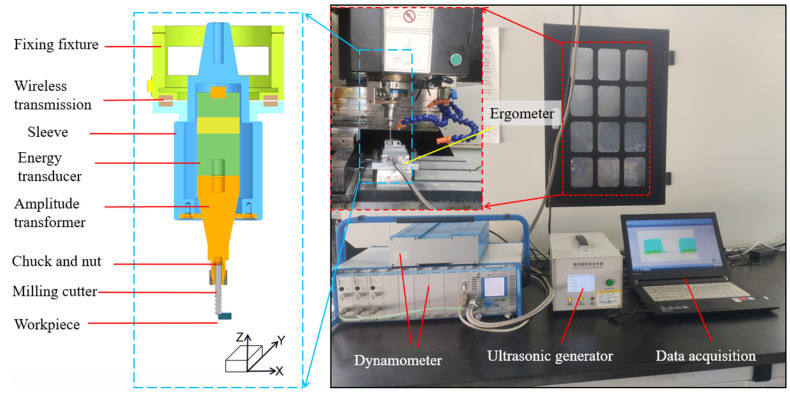
Milling test platform.

The milling test used a CNC machining center (Henfux-HFM 700L, Henfux Group Co., Ltd., Shenzhen, China), and the test site is shown in Figure 1. In UAM testing, the vibration frequency generated by the ultrasonic device is 24.8 kHz, and the ultrasonic power supply transmits energy to the energy transducer. The energy transducer subsequently converted the electrical signal into high-frequency vibration and transmitted it to the amplitude transformer, thereby achieving vibration machining. The energy transducer–amplitude transformer assembly was installed in the ultrasonic cutter and rotated with the spindle at high speed. The energy transmission between the ultrasonic power supply and the energy transducer was achieved wirelessly through coil induction, addressing both the “non-rotation” issue of the ultrasonic power supply and the “rotation” issue of the energy transducer-amplitude transformer assembly. The cutting force measurement and acquisition systems were installed under the workpiece to obtain real-time information about machining and inspection data (the milling force-acquisition system type 9119AA2 and the signal amplifier type 5080A; Swiss Kistler Instruments Co., Ltd., Winterthur, Switzerland), which converted mechanical pressure signals into electrical signals through the piezoelectric effect and input the signals into the signal amplifier for data generation. The dynamometer sampling frequency is set to 2000 Hz. The roughness and surface morphology were measured using a digital microscope with an ultra-deep field of view (Easy Zoom 5 3D, Motic China Group Co., Ltd., Xiamen, China). This study used an afour-blade integral universal hard alloy circular arc end mill (Zhuzhou Cemented Carbide Cutting Tools Co., Ltd., Zhuzhou, China, model: TM-3R-D6.0 R0.5). The milling cutter specifications were as follows: diameter of 6 mm, blade length of 20 mm, total length of 60 mm, and an AlCrXN coating. The overhang of the tool after clamping is 36 mm.

### 2.2. Experiment Scheme Design

#### 2.2.1. Single Factor Variable Experimental Design

To explore the influences of different cutting parameters on the milling force under ultrasonic vibration, the spindle speed *n*, feed per tooth *f*_z_, milling depth *a*_p_, and ultrasonic amplitude *A* were used as variables to conduct a univariate test for UAM of Ti alloys. The machining method was end milling, and each group of tests was repeated three times to reduce error and improve accuracy. Based on similar studies and machining practices [27,28], the spindle speed was determined as 1000–3000 r/min, the feed per tooth was 0.01–0.05 mm/z, the radial milling depth was 0.1–0.5 mm, and the ultrasonic amplitude was 1–5 μm. For the purpose of comparative research, each group of single-factor experiments was conducted using CM as a reference, with an amplitude of 0 and other parameters remaining unchanged. The process parameters are shown in Table 3.

#### 2.2.2. Multivariate Experimental Design

The variables used in the multivariate orthogonal test were also the spindle speed *n* (denoted as A), feed per tooth *f*_z_ (denoted as B), milling depth *a*_p_ (denoted as C), and ultrasonic amplitude *A* (denoted as D), and four levels were set for each variable, as shown in Table 4.

**Figure 2:**
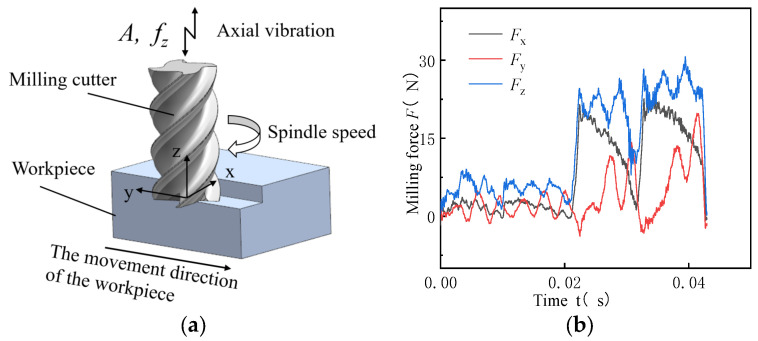
The trend of milling mode and force in ultrasonic vibration machining. (**a**) Schematic of axial ultrasonic vibration milling; (**b**) Trend of milling force in one cycle of ultrasonic vibration machining.

The milling force measured using the dynamometer was a dynamic force (Figure 2). As the milling force measured during milling is a transient quantity that alternatively changes between positive and negative values, it is inappropriate to directly select a fixed value from the dynamic curve as a reference. If the average milling force value is used as the milling force value, positive and negative values offset each other. Consequently, the variations in the milling force during cutting cannot be reflected. Therefore, the mean square root of the milling force was considered as the milling force during cutting for comparison in this study.

## 3. Results

### 3.1. Univariate Test of Milling Force

#### 3.1.1. Effects of Spindle Speed on Milling Force

**Figure 3:**
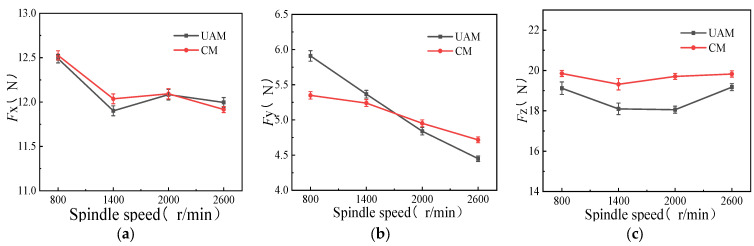
Effects of spindle speed on milling force (*f*_z_ = 0.01 mm/z, *a*_p_ = 0.2 mm, *A* = 3 μm). (**a**) *F_x_* as a function of spindle speed; (**b**) *F_y_* as a function of spindle speed; (**c**) *F_z_* as a function of spindle speed.

Comparative experiments were conducted for CM and UAM under different spindle speeds of 800, 1400, 2000, and 2600 r/min, with other variables remaining constant such as Table 3 (*f*_z_ = 0.01 mm/z, *a*_p_ = 0.2 mm, *A* = 3 μm). As shown in Figure 3, under constant feed per tooth, milling depth, and amplitude, UAM and CM showed the same decreasing trend of milling force on three axes with the increase in the spindle speed. As the spindle speed increased, the contact time between the cutter and the workpiece during cutting was shortened. Thus, the plastic deformation duration of the cutting area decreased, resulting in a decrease in the cutting force. Meanwhile, the flow rate of the metal material during cutting also increased, leading to more uniform plastic deformation and decreased cutting force.

Comparative analysis of the two milling modes revealed that when the spindle speed was less than 2000 r/min, the milling force generated using UAM was generally smaller than that generated using CM. The maximum difference in the milling force between the two milling modes was 0.14 N, and the milling force was reduced by 1.16% as the spindle speed increased from 800 to 2000 r/min (Figure 3a, *x*-axis). Further, when the spindle speed was more than 2000 r/min, the milling force generated using CM was smaller than that using UAM. The milling force on the *y*-axis exhibited an opposite trend (Figure 3b). Specifically, when the spindle speed was less than 1600 r/min, the milling force generated using UAM was larger than that using CM, the maximum difference of the milling force between the two milling modes was 0.56 N, and the milling force was reduced by 9.48% as the spindle speed increased from 800 to 1600 r/min. Moreover, when the spindle speed was more than 1600 r/min, the milling force generated using CM was larger than that using UAM, indicating that the effects of UAM on the milling force on the *y*-axis were smaller than that of CM. The milling force generated using UAM was always smaller than that generated using CM (Figure 3c, *z*-axis). In this case, when the spindle speed was less than 2000 r/min, the difference in the milling force between the two modes gradually increased with the increase in the spindle speed. When the spindle speed increased from 800 to 2000 r/min, the reduction rate of the milling force under ultrasonic vibration increased from 3.97% to 8.6%. Furthermore, when the spindle speed was more than 2000 r/min, the difference in the milling force between the two modes gradually decreased.

Hence, when UAM was used, the milling force was generated by the contact between the cutter and the workpiece; when the cutter and the workpiece were separated, the milling force disappeared. Hence, the milling force generated using UAM was relatively small, and the effects of UAM were relatively significant on the *x*- and *z*-axes. Therefore, the spindle speed should be set to 2000 r/min to minimize the milling force under UAM.

#### 3.1.2. Effects of Feed Rate per Tooth on Milling Force

**Figure 4:**
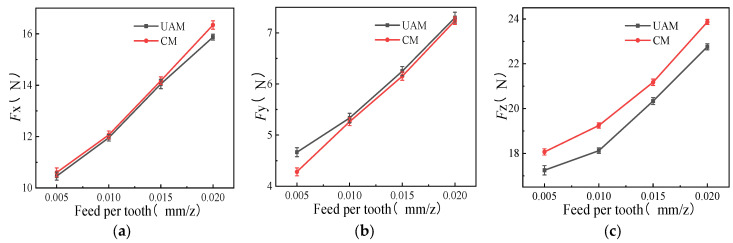
Effects of feed per tooth on milling force (*n* =1400 r/min, *a*_p_ = 0.2 mm, *A* = 3 μm). (**a**) *F_x_* as a function of feed per tooth; (**b**) *F_y_* as a function of feed per tooth; (**c**) *F_z_* as a function of feed per tooth.

Comparative experiments were conducted for CM and UAM under different feed rates per tooth of 0.005, 0.010, 0.015, and 0.020 mm/z, with other variables remaining constant such as Table 3 (*n* =1400 r/min, *a*_p_ = 0.2 mm, *A* = 3 μm). As the feed per tooth increased, both milling modes showed significantly increasing milling force on the *x*-, *y*-, and *z*-axes (Figure 4). This was because the cutting area of the cutter increased and the amount of materials removed increased with the increase in the feed per tooth. Additionally, the cutter wear increased due to the increase in plastic deformation of the material, hence requiring more cutting force to ensure cutting quality and machining efficiency. Therefore, the milling force showed an overall increasing trend.

The milling force generated using UAM was smaller than that using CM on the *x*- and *z*-axes under the same milling conditions, but the difference in the milling force between the two modes on the *x*-axis was not significant, showing only a 1–2% decrease. Further, on the *z*-axis, when the feed per tooth was 0.01 mm/z, the milling force generated using UAM was reduced by 5.97% compared with that using CM. However, the effects of UAM on the *y*-axis were not greater than that of CM, and the milling force generated using UAM was similar to that using CM.

The results of this study revealed that the pulse cutting generated using UAM shortened the effective cutting time of the cutter, resulting in reduced friction between the cutter, workpiece, and cutting chips. Also, the effects of UAM on the *z*-axe were relatively significant, however, there is no essential difference between UAM and CM in the *x*-axis and *y*-axis directions, meaning that longitudinal vibration does not have a significant improvement effect on the cutting force of the *x*-axis and *y*-axis.

#### 3.1.3. Effects of Milling Depth on Milling Force

**Figure 5:**
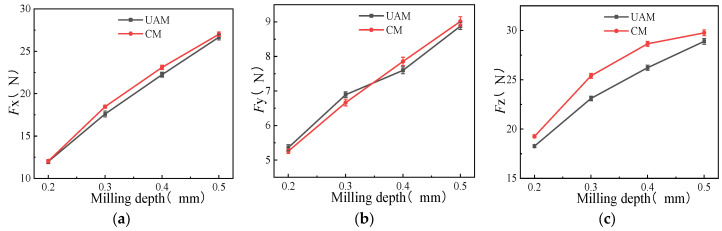
Effects of milling depth on milling force (*n* =1400 r/min, *a*_p_ = 0.2 mm, *A* = 3 μm). (**a**) *F_x_* as a function of milling depth; (**b**) *F_y_* as a function of milling depth; (**c**) *F_z_* as a function of milling depth.

Comparative experiments were conducted for CM and UAM under different milling depths of 0.2, 0.3, 0.4, and 0.5 mm, with other variables remaining constant, such as Table 3 (*n* =1400 r/min, *a*_p_ = 0.2 mm, *A* = 3 μm). As shown in Figure 5, both milling modes showed increasing milling force on the *x*-, *y*-, and *z*-axes with the increase in the milling depth. The increment tends of milling force generated using UAM and CM increased first and then decreased on the *x*- and *z*-axes, with a milling depth of 0.3–0.4 mm as the demarcation point. The maximum reduction rate of the milling force under ultrasonic vibration on the *x*- and *z*-axes was 4.34% and 9.41%, respectively. A milling depth of 0.3–0.4 mm was also taken as the demarcation point on the *y*-axis, but the ability to reduce the milling force of UAM was not as good as that of CM in the beginning but subsequently exceeded that of CM.

In summary, the milling depth cannot be too large during ultrasonic vibration milling, as a too-large milling depth not only leads to linear growth of the milling force but also reduces the advantages of UAM. Therefore, a milling depth of about 0.4 mm should be used to ensure good ultrasonic vibration performance during UAM milling.

#### 3.1.4. Effects of Ultrasonic Amplitude on Milling Force

**Figure 6:**
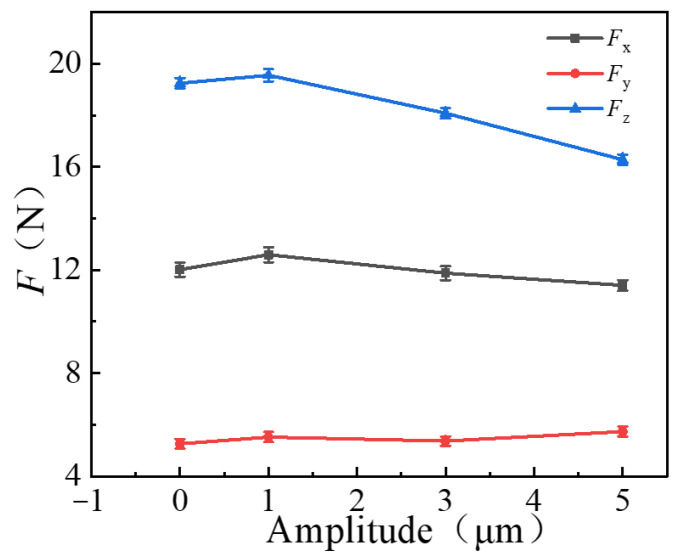
Effects of amplitude on triaxial milling force on three axes. (*n* = 1400 r/min, *a*_p_ = 0.2 mm, *f*_z_ = 0.01 mm/z).

Comparative experiments were conducted for CM and UAM under different ultrasonic amplitudes of 0, 1, 3, and 5 μm, with other variables remaining constant, such as Table 3 (*n* =1400 r/min, *a*_p_ = 0.2 mm, *f*_z_ =0.01 mm/z). As shown in Figure 6, the milling forces *F_x_* and *F_z_* showed a decreasing trend with the increase in ultrasonic amplitude, but when the amplitude is 1, the cutting force of UAM is slightly larger than that of CM, indicating that a smaller amplitude is not positive for reducing the cutting force. The milling force generated using UAM on the *z*-axis reduced from 19.25 to 16.27 N when the amplitude was 5 μm, with a reduction rate of 15.48%. The milling force generated using UAM on the *x*-axis reduced from 12.04 to 11.40 N, with a reduction rate of 5.32%, compared with CM (*A* = 0 μm). However, the milling force *F_y_* slightly increased with the increase in amplitude. Overall, the milling force on the *x*- and *z*-axes significantly improved as the ultrasonic amplitude increased, but the milling force on the *y*-axis did not vary significantly from CM, indicating a relatively weak improvement on the *y*-axis under increased ultrasonic amplitude. 

### 3.2. Orthogonal Test of Milling Force

This study involved an orthogonal test with four factors and four levels (assuming the fifth factor is null), that is, L_16_ (4^4^), based on the standard orthogonal table of five factors and four levels. Sixteen groups of tests were conducted; the test scheme and results were as depicted in Table 5.

#### 3.2.1. Range Analysis of *F_x_*

**Figure 7:**
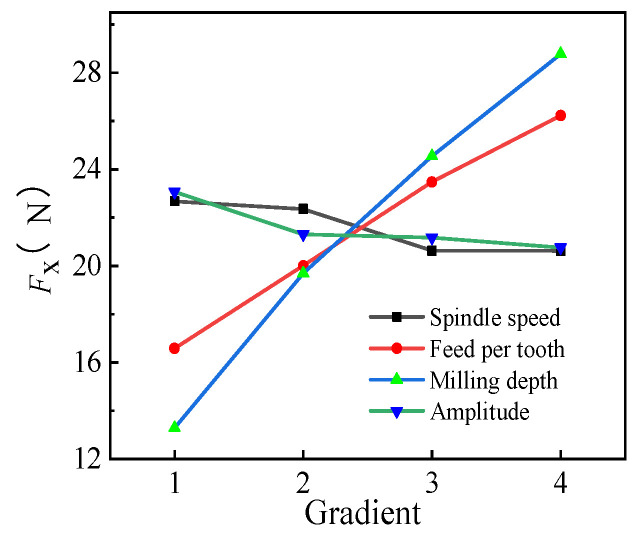
The trend of *F_x_* under different horizontal parameters.

The *F_x_* range is depicted in Table 6, and its trend is shown in Figure 7. The milling force *F_x_* on the *x*-axis increased with the increase in the feed per tooth *f*_z_ and milling depth *a*_p_, t, but *F_x_* only indicated slight fluctuations with the increase in *n* and *A*. Based on the range of *F_x_*, R(*a*_p_) = 15.495 > R(*f*_z_) = 9.648 > R(*A*) = 2.3 > R(*n*) = 2.074. Therefore, the effects on the milling force in the milling plane were in the order *a*_p_ > *f*_z_ > *A* > *n*. The optimized combination was found to be A_3_B_1_C_1_D_5_ (*n* = 2000 r/min, *f*_z_ = 0.005 mm/z, *a*_p_ = 0.2 mm, and *A* = 5 μm) based on the aforementioned analysis, as the combination of the machining parameters could effectively reduce the milling force on the *x*-axis. *F_x_* was 9.922 N under the optimized parameter combination based on the verification tests conducted using the aforementioned data, which was lower than all the values obtained in the orthogonal test. The maximum and minimum reduction rates of *F_x_* were 70.81% and 11.77%, respectively, validating the optimized combination obtained in the orthogonal test.

#### 3.2.2. Range Analysis of *F_y_*

**Figure 8:**
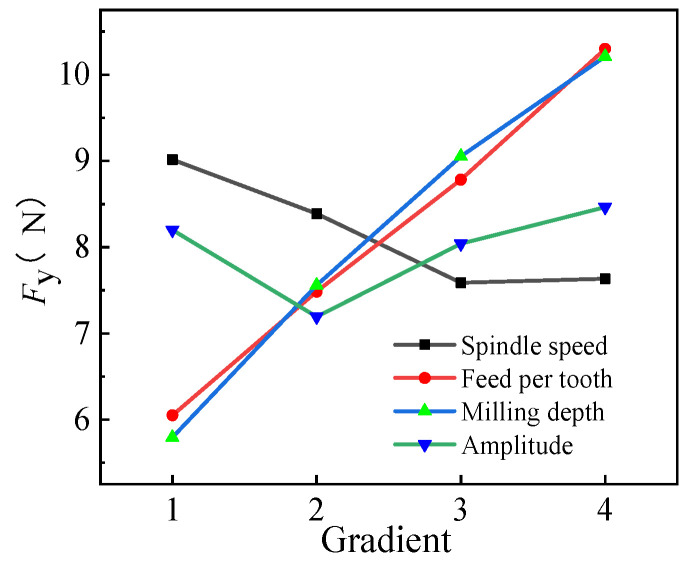
Trend of *F_y_* under different horizontal parameters.

The range of *F_y_* is depicted in Table 7 and its trend is shown in Figure 8. As observed, R(*a*_p_) = 4.420 > R(*f*_z_) = 4.251 > R(*n*) = 1.428 > R(*A*) = 0.552. The effects on the milling force in the milling plane were in the order *a*_p_ > *f*_z_ > *n* > *A*. The optimized combination was A_3_B_1_C_1_D_2_ (*n* = 2000 r/min, *f*_z_ = 0.005 mm/z, *a*_p_ = 0.2 mm, and *A* = 1 μm) based on the aforementioned analysis, as the combination of the machining parameters could effectively reduce the milling force on the *y*-axis.

According to verification tests using the aforementioned data, *F_y_* was 4.791 N under the optimized parameter combination, which was lower than most of the values obtained in the orthogonal test (except for group 1 with slightly higher *F_y_*). The maximum and minimum reduction rates of *F_y_* were 65.17% and 15.52%, respectively, validating the optimized combination obtained in the orthogonal test.

#### 3.2.3. Range Analysis of *F_z_*

**Figure 9:**
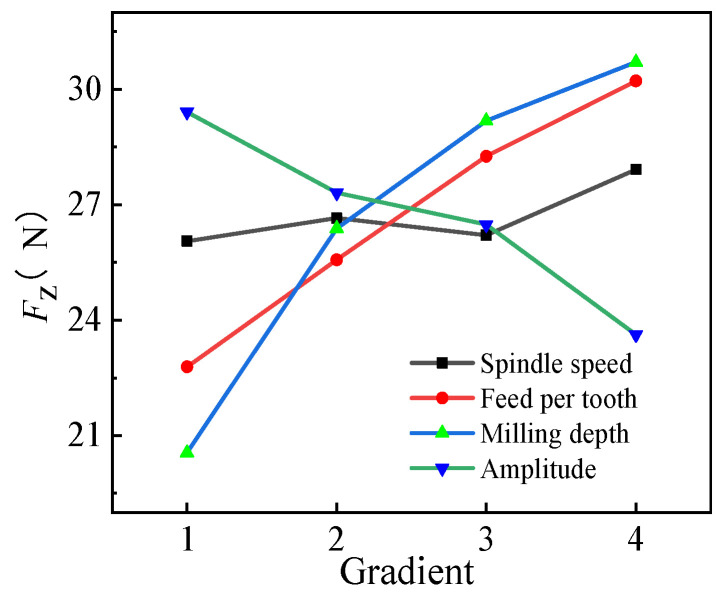
The trend of *F_z_* under different horizontal parameters.

The range of *F_z_* is depicted in Table 8, and its trend is shown in Figure 9. As observed, R(*a*_p_) = 10.152 > R(*f*_z_) = 7.431 > R(*A*) = 5.791 > R(*n*) = 1.868. Therefore, the effects on the milling force in the milling plane were in the order *a*_p_ > *f*_z_ > *A* > *n*. Meanwhile, the ranges of ultrasonic amplitude and feed per tooth were similar, indicating increased effects of ultrasonic amplitude on the *z*-axis. Therefore, the optimized combination was A_1_B_1_C_1_D_4_ (*n* = 800 r/min, *f*_z_ = 0.005 mm/z, *a*_p_ = 0.2 mm, and *A* = 5 μm), as the combination of the machining parameters could minimize the milling force on the *z*-axis.

The experiments revealed that *F_z_* was 15.488 N under the optimized parameter combination, which was lower than all the values obtained in the orthogonal test. The maximum and minimum reduction rates of *F_z_* were 55.68% and 17.66%, respectively, validating the optimized combination obtained in the orthogonal test.

### 3.3. Surface Roughness

Surface roughness refers to the irregularity of surface micro-texture or the unevenness of surface elevation. It is usually used to describe the smoothness or roughness of the machined surface and is an important technical index to evaluate workpiece surface quality [29]. Roughness directly affects the performance, durability, and lifetime of a workpiece. Surface roughness is usually characterized by the maximum height of the profile *R*_z_, arithmetic means deviation *R*_a_, and mean square root roughness *R*_q_.

**Figure 10:**
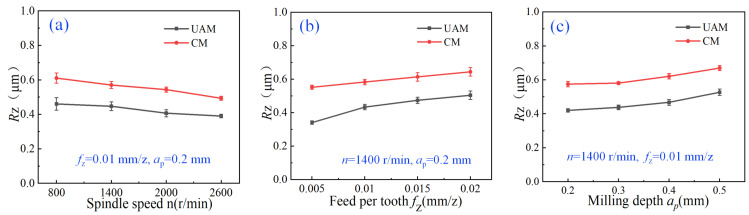
Trend of roughness under different parameters. (**a**) The relationship between spindle speed and surface roughness; (**b**) The relationship between feed per tooth and surface roughness; (**c**) The relationship between milling depth and surface roughness.

In this study, according to Chinese national standards” Geometrical product specifications (GPS)—Surface texture: Profile method—Terms, definitions and surface texture parameters (GB T 3505-2009, Equivalent to ISO 4287: 1997, IDT)” [30], the maximum height of the profile *R*_z_ was used to characterize roughness. To reduce measurement errors and inaccuracies, the roughness values were obtained from three different areas on the machined surface. These values were then averaged to generate the final results for analysis and research. The test results and the variation trend of surface roughness are illustrated in Figure 10. All the roughness values of UAM were lower than those of CM. The cutting edge moved quickly and precisely on the workpiece surface under high-frequency vibration during ultrasonic vibration machining, which produced small cuts and effectively removed burrs and particles from the workpiece surface, thus reducing the surface roughness.

#### 3.3.1. Effects of Spindle Speed on Roughness

In this experiment, *f*_z_ and *a*_p_ remained constant at 0.01 mm/z and 0.2 mm, respectively. When *n* was relatively low, the cutting speed was relatively slow, the cutting chips were relatively large, and the surface roughness was relatively high. As *n* increased, the cutting speed increased, and the cutting chips became finer resulting in a smoother surface and reduced surface roughness. As shown in Figure 10a, when *n* increased from 800 to 2600 r/min, the roughness of CM and UAM reduced by 18.03% and 17.02%, respectively; when *n* was 2000 r/min, the roughness of UAM was reduced by the largest amount of 0.14 μm, with a reduction rate of 25.93%, compared with that of CM.

#### 3.3.2. Effects of Feed per Tooth on Roughness

In this experiment, *n* and *a*_p_ remained constant at 1400 r/min and 0.2 mm, respectively. As *f*_z_ increased, the surface roughness increased. When *f*_z_ was relatively low, the force generated between the cutter and the machined material was relatively small, and the cutting particles were relatively large, resulting in reduced surface roughness. As *f*_z_ increased, the force generated between the cutter and the machined material increased, and the size of the cutting particles increased, resulting in increased surface roughness. As shown in Figure 10b, when *f*_z_ was 0.005 mm/z, the roughness of UAM was reduced by the largest amount of 0.2 μm, with a reduction rate of 36.36%, compared with that of CM.

#### 3.3.3. Effects of Milling Depth on Roughness

In this experiment, *n* and *f*_z_ remained constant at 1400 r/min and 0.01 mm/z, respectively. When *a*_p_ was relatively small, negligible contact was observed between the cutter and the machined material. Also, not much heat and stress were generated during milling, which was conducive to ensuring surface quality and prolonging the cutter’s lifetime. However, the contact area increased with the increase in *a*_p_, which resulted in generating a large quantity of heat between the cutter and the machined material. This led to surface burns, stress concentration, and other surface defects, thus affecting surface quality. As shown in Figure 10c, as *a*_p_ increased, although the roughness of the two milling modes tended to increase almost in parallel, the roughness of UAM was always lower than that of CM (0.15 μm), with a maximum reduction rate of 26.32%, indicating better surface quality.

### 3.4. Surface Morphology

**Figure 11:**
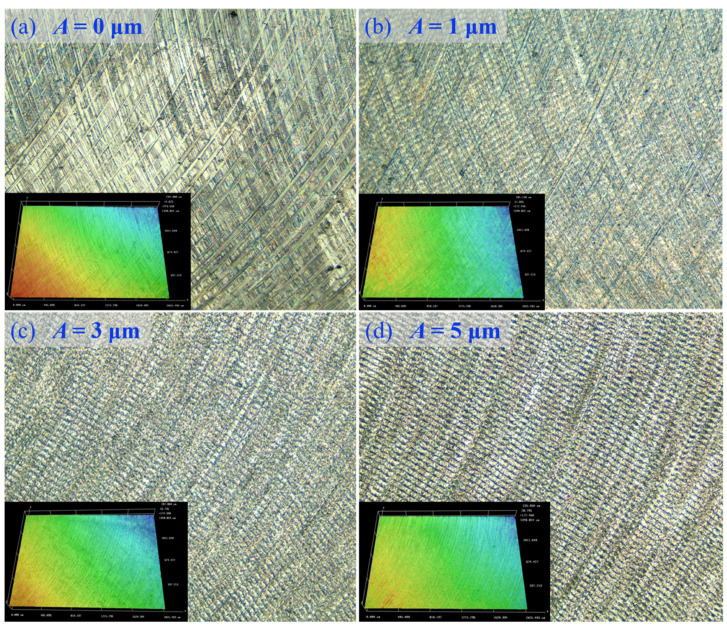
Surface morphologies of Ti alloys under different amplitudes. (**a**) The surface morphology at CM (amplitude *A* = 0 μm); (**b**) The Surface morphology at amplitude *A* = 1 μm; (**c**) The Surface morphology at amplitude *A* = 3 μm; (**d**) The Surface morphology at amplitude *A* = 5 μm.

The effects of ultrasonic amplitude on surface morphology were tested at *n* = 1400 r/min, *f*_z_ = 0.01 mm/z, and *a*_p_ = 0.2 mm to explore the variation in surface morphology under UAM of Ti alloys. The ultrasonic amplitudes are 0, 1, 3, and 5 μm respectively. The surface morphology test results are shown in Figure 11, and the measured surface roughness values are Rz_(0)_ = 0.65, Rz_(1)_ = 0.18, Rz_(3)_ = 0.25, and Rz_(5)_ = 0.62 μm, respectively.

As shown in Figure 11, different amplitudes had varying effects on the surface morphology. When *A* was 0 μm (under CM), the cutting chips were attached to the Ti alloy surface, and hence different depths of scratches were generated on the surface under the extrusion of the milling cutter, affecting surface integrity. Further, the milling surface had obvious cutting marks, and the surface morphology was disordered, the surface roughness is Rz_(0)_ = 0.65 μm. As the ultrasonic vibration amplitude increased, the micro-texture of the Ti alloy surface gradually appeared. The surface condition was better than that depicted in Figure 11a when *A* was 1 μm (Figure 11b), and the cutting marks on the surface were further reduced, surface roughness Rz_(1)_ = 0.18 μm. The surface condition was the best when *A* was 3 μm (Figure 11c), and no cutting marks were observed on the surface, the micro-texture was distributed uniformly, and fish scale machining meshes were distributed on the machined surface. Further, the micro-texture was relatively uniform and regular, and the surface morphology was clear, with surface roughness Rz_(3)_ = 0.25 μm. When the amplitude increases to 5 μm. As shown in Figure 11d, the micro-texture of the processed surface is clearer, and deeper grooves appear around the scaly processing patterns. Although the micro-texture features of the morphology are strengthened, the surface roughness value increases to Rz_(5)_ = 0.62 μm, resulting in a decrease in surface quality.

From the above analysis, it can be seen that as the amplitude increases, the quality of surface micro-textures in ultrasonic-assisted milling shows a trend of first increasing and then decreasing. Surface morphology should not be used as a separate criterion for judgment, but must be combined with roughness values to determine the combination of process parameters. In this example, when the amplitude is 1 μm. Although the surface roughness is the smallest, the processed surface has a poor appearance, and the micro-texture features are not obvious enough; when the amplitude is 5 μm. Although the micro texture features are the strongest, the surface roughness also increases significantly; when the amplitude is 3 μm. It can achieve ideal surface micro-texture and surface roughness simultaneously.

## 4. Conclusions

The cutting force and surface integrity of the Ti alloy TC4 machined using UAM and CM were investigated in this study. The following conclusions were drawn:

1. The univariate test revealed that ultrasonic amplitude had the maximum effect on the milling force on the *z*-axis, and the milling force generated using UAM was reduced by 15.48% compared with that generated using CM.
2. The multivariate range analysis revealed that *a*_p_ and *f*_z_ were the major factors affecting the cutting force. Compared with CM, UAM had the minimum milling force reduction rates of 11.77%, 15.52%, and 17.66% and maximum reduction rates of 70.81%, 65.17%, and 55.68% on the *x*-, *y*-, and *z*-axes, respectively.
3. The machined surface roughness was lower and the surface quality was better under UAM. Compared with CM, UAM had the maximum surface roughness reduction rates of 25.93%, 36.36%, and 26.32% in terms of *n*, *f*_z_, and *a*_p_, respectively. The impact of high-frequency vibration of the cutting edge during UAM milling led to precision machining of the workpiece surface and smaller cutting force generated by contact, thus effectively reducing roughness.
4. Compared with CM, longitudinal ultrasonic vibration milling showed periodic “separation—contact,” resulting in fish scale machining meshes on the machined surface. Under appropriate processing parameters, this structure was regularly and uniformly distributed on the machined surface, leading to a high workpiece surface finish and smoothness.

## Figures and Tables

**Table 1:** Elemental composition of Ti alloy TC4 (mass fraction, %).

Al	V	Ti	Fe	C	N	H	O	Individual	Total
5.5–6.8	3.5–4.5	Rest	0.30	0.10	0.05	0.015	0.20	0.10	0.40

**Table 2:** Thermodynamic parameters of Ti alloy TC4.

Density (kg/m^3^)	Elastic Modulus (GPa)	Yield Strength (MPa)	Tensile Strength (MPa)	Hardness (HRC)	Thermal Conductivity (W/mK)	Poisson’s Ratio (μ)
4440	110	860	905	30	7.995	0.34

**Table 3:** Univariate test.

Sample	Spindle Speed *n* (r/min)	Feed Rate per Tooth *f*_z_ (mm/z)	Cutting Depth *a*_p_ (mm)	Amplitude *A* (μm)
1–4	800, 1400, 2000, 2600	0.01	0.2	3
5–8	1400	0.005, 0.01, 0.015, 0.02	0.2	3
9–12	1400	0.01	0.2, 0.3, 0.4, 0.5	3
13–16	1400	0.01	0.2	0, 1, 3, 5

**Table 4:** Parameters in the orthogonal tests.

Level	Spindle Speed *N*(A) (r/min)	Feed per Tooth *f*_z_(B) (mm/z)	Milling Depth *a*_p_(C) (mm)	Amplitude *A*(D) (μm)
1	800	0.005	0.2	0
2	1400	0.010	0.3	1
3	2000	0.015	0.4	3
4	2600	0.020	0.5	5

**Table 5:** Scheme and results of orthogonal tests.

No.	Spindle Speed *n*(A) (r/min)	Feed per Tooth *f*_z_(B) (mm/z)	Milling Depth *a*_p_(C) (mm)	Amplitude *A*(D) (μm)	*F_x_* (N)	*F_y_* (N)	*F_z_* (N)
1	800	0.005	0.2	0	11.245	4.508	18.810
2	800	0.01	0.3	1	18.719	7.364	25.226
3	800	0.015	0.4	3	26.838	10.427	29.873
4	800	0.02	0.5	5	33.988	13.757	30.290
5	1400	0.005	0.3	3	15.314	5.800	22.001
6	1400	0.01	0.2	5	11.357	5.671	16.287
7	1400	0.015	0.5	0	32.697	10.976	34.943
8	1400	0.02	0.4	1	30.067	11.106	33.368
9	2000	0.005	0.4	5	17.548	6.545	21.713
10	2000	0.01	0.5	3	26.205	8.773	28.974
11	2000	0.015	0.2	1	14.225	5.839	22.030
12	2000	0.02	0.3	0	24.560	9.186	32.116
13	2600	0.005	0.5	1	22.247	7.348	28.613
14	2660	0.01	0.4	0	23.753	8.132	31.778
15	2600	0.015	0.3	5	20.164	7.891	26.193
16	2600	0.02	0.2	3	16.328	7.155	25.087

**Table 6:** Range analysis of *F_x_*.

	Spindle Speed *n* (r/min)	Feed Rate per Tooth *f*_z_ (mm/z)	Milling Depth *a*_p_ (mm)	Amplitude *a* (μm)
*K*1	22.679	16.588	13.289	23.064
*K*2	22.359	20.008	19.689	21.315
*K*3	20.623	23.481	24.552	21.171
*K*4	20.634	26.236	28.784	20.764
Range	2.074	9.648	15.495	2.300

**Table 7:** Range analysis of *F_y_*.

	Spindle Speed *n* (r/min)	Feed Rate per Tooth *f*_z_ (mm/z)	Milling Depth *a*_p_ (mm)	Amplitude *A* (μm)
*K*1	9.014	6.050	5.793	8.200
*K*2	8.388	7.485	7.560	7.194
*K*3	7.586	8.783	9.053	8.039
*K*4	7.632	10.301	10.213	8.466
Range	1.428	4.251	4.420	0.552

**Table 8:** Range analysis of *F_z_*.

	Spindle Speed *n* (r/min)	Feed Rate per Tooth *f*_z_ (mm/z)	Milling Depth *a*_p_ (mm)	Amplitude *A* (μm)
*K*1	26.050	22.784	20.553	29.412
*K*2	26.650	25.566	26.384	27.309
*K*3	26.208	28.260	29.183	26.484
*K*4	27.918	30.215	30.705	23.621
Range	1.868	7.431	10.152	5.791

## Data Availability

All relevant data can be obtained in this article.
